# Oxidative Stress and Inflammatory Markers in Abdominal Aortic Aneurysm

**DOI:** 10.3390/antiox10040602

**Published:** 2021-04-14

**Authors:** David Sánchez-Infantes, Meritxell Nus, Miquel Navas-Madroñal, Joan Fité, Belén Pérez, Antonio J. Barros-Membrilla, Begoña Soto, José Martínez-González, Mercedes Camacho, Cristina Rodriguez, Ziad Mallat, María Galán

**Affiliations:** 1 Department of Basic Sciences of Health, Area of Biochemistry and Molecular Biology, University Rey Juan Carlos, 28922 Alcorcón, Spain; dsanchez@igtp.cat; 2 Centro de Investigación Biomédica en Red-Fisiopatología de la Obesidad y Nutrición (CIBEROBN), ISCIII, 28029 Madrid, Spain; 3 Division of Cardiovascular Medicine, University of Cambridge, Cambridge CB2 0QQ, UK; mn421@cam.ac.uk (M.N.); zm255@medschl.cam.ac.uk (Z.M.); 4 Centro de Investigación Biomédica en Red de Enfermedades Cardiovasculares (CIBERCV), ISCIII, 28029 Madrid, Spain; jose.martinez@iibb.csic.es (J.M.-G.); mcamacho@santpau.cat (M.C.); crodriguezs@santpau.cat (C.R.); 5 Institut de Recerca del Hospital de la Santa Creu i Sant Pau, 08041 Barcelona, Spain; mnavasm7@gmail.com; 6 Instituto de Investigación Biomédica Sant Pau (IB Sant Pau), 08025 Barcelona, Spain; 7 Servicio de Angiología, Cirugía Vascular y Endovascular, Hospital de la Santa Creu i Sant Pau, 08041 Barcelona, Spain; jfite@santpau.cat (J.F.); bsoto@santpau.cat (B.S.); 8 Faculty of Medicine, Universidad Autónoma de Barcelona, Bellaterra, 08193 Barcelona, Spain; Belen.Perez@uab.cat; 9 Unidad Funcional de Patología de la Aorta (UPA), Servicio de Cardiología, Hospital de la Santa Creu i Sant Pau, 08041 Barcelona, Spain; abarros@santpau.cat; 10 Instituto de Investigaciones Biomédicas de Barcelona-Consejo Superior de Investigaciones Científicas (IIBB-CSIC), 08036 Barcelona, Spain

**Keywords:** abdominal aorta aneurysm, biomarkers, inflammation, oxidative stress, prognosis

## Abstract

Abdominal aortic aneurysm (AAA) is increasing due to aging of the population and is a major cause of death among the elderly. Ultrasound screening programs are useful in early diagnosis, but aneurysm size is not always a good predictor of rupture. Our aim was to analyze the value of circulating molecules related to oxidative stress and inflammation as new biomarkers to assist the management of AAA. The markers were quantified by ELISA, and their expression in the aneurysmal wall was studied by real-time PCR and by immunostaining. Correlation analysis of the studied markers with aneurysm diameter and peak wall stress (PWS), obtained by finite element analysis, and multivariate regression analysis to assess potential confounding factors were performed. Our study shows an extensive inflammatory infiltration in the aneurysmal wall, mainly composed by T-cells, macrophages and B-cells and altered levels of reactive oxygen species (ROS), IgM, IgG, CD38, GDF15, S100A4 and CD36 in plasma and in the aneurysmal tissue of AAA patients compared with controls. Circulating levels of IgG, CD38 and GDF15 positively correlated with abdominal aortic diameter, and CD38 was correlated with PWS. Our data show that altered levels of IgG, CD38 and GDF15 have potential diagnostic value in the assessment of AAA.

## 1. Introduction

Rupture of abdominal aortic aneurysm (AAA) is a life-threatening condition with a high mortality rate [1]. The implementation of ultrasound screening programs to identify small AAAs has improved the current diagnosis and management of this condition; however, rates of expansion of AAAs during follow-up vary for intra and inter-patients [2,3]. Although the risk of rupture increases exponentially with maximal anterior–posterior aortic diameter, aneurysm size is not always a good predictor since small aneurysms can lead to rupture and a proportion of large AAA remain stable overtime [4,5]. The identification of circulating markers of AAA progression will complement the measurement of aortic diameter and will help to improve the identification of patients at risk of aneurysm rupture [4].

Different pathological mechanisms converge on AAA development, such as vascular oxidative stress, inflammation, extracellular matrix (ECM) remodeling, altered cell signaling and increased apoptosis in vascular smooth muscle cells (VSMCs) [1,6]. Chronic inflammation plays a central role promoting proteinases-induced tissue destruction and suppressing tissue repair in AAA [7]. Further, the implication of local oxidative stress in the pathogenesis of AAA is also well documented [8,9,10]. Human AAA tissue is characterized by an early infiltration of innate and adaptive inflammatory cells, which occurs before detectable ECM destruction and aortic diameter expansion [11,12,13]. This inflammatory environment critically contributes to vascular oxidative stress, which in turn could exacerbate the recruitment of inflammatory cells in a reciprocal feedback loop [13].

In order to identify new circulating biomarkers and determine their potential prognostic value in aneurysm progression, we selected a battery of immune-inflammatory and oxidative stress markers, involved in the degenerative events associated with the pathophysiology of AAA. In particular, we focused on the transmembrane enzyme CD38, growth differentiation factor 15 (GDF15), the member of the S100 calcium-binding protein family, S100A4, and the lipid scavenger receptor CD36 proteins that are expressed on the surface of lymphocytes, macrophages, endothelial cells and VSMC. All of them are involved in oxidative stress generation and in inflammatory processes underlying vascular remodeling and contribute to phenotypic changes and cellular apoptosis under different pathological conditions [14,15,16,17,18,19,20,21,22,23]. In the current paper, we analyzed vascular expression and circulating levels of IgM, IgG, reactive oxygen species (ROS), CD38, GDF15, S100A4 and CD36 in samples from patients with AAA vs. healthy controls and for the first time, assessed their potential association with two well-established parameters for predicting the risk of rupture, aneurysm diameter and peak wall stress (PWS). 

## 2. Materials and Methods

### 2.1. Human Samples

Human abdominal aneurysmal aortas (*n* = 80) and blood samples (*n* = 94) were obtained from patients diagnosed with infrarenal AAA and undergoing open repair or endovascular surgery repair for AAA at the Hospital de la Santa Creu i Sant Pau (HSCSP; Barcelona, Spain). The diagnosis of AAA was confirmed by computed tomography (CT) scan. Patients with AAA and with negative histories of rheumatological, immunological diseases, aortitis or genetic syndromes such as Marfan disease were included in the study. Other exclusion criteria were juxtarenal aneurysms and mycotic aneurysms. The plasma samples were collected prior to anesthesia on the same day of the surgical intervention. Healthy abdominal aortas (*n* = 15) came from multiorgan donors. The Ethics Committee of the HSCSP approved the use of the discarded human tissue and participation in the study of patients and controls was based upon informed consent. Research was conducted in accordance with the Declaration of Helsinki of 1975. Abdominal aorta segments and plasma samples were obtained according to standard operating procedures and ethical guidelines. No signs of AAA or evidence of atherosclerotic plaques in the abdominal aorta were found in samples of control subjects. For RNA and protein studies part of the tissue samples were collected, snap-frozen and stored at −80 °C and part were fixed and embedded in paraffin for subsequent immunostaining assays. The blood samples from healthy donors were provided by the Banc de Sang i Teixits (BST, Barcelona, Spain) according to the following inclusion criteria: healthy subjects aged 55–65 years, non-hypertensive or with pharmacologically controlled hypertension and without previously reported cardiovascular complications (*n* = 46).

### 2.2. ELISA Kits

The circulating levels of soluble IgM (ab214568, Abcam, UK), IgG (ab195215), soluble CD38 (KIT10818-1, Sino Biological Inc., Wayne, PA, USA), soluble CD36 (ABE-196-02, Nordic BioSite, Täby, Sweden), S100A4 (CSB-EL020632HU, Cusabio Biotech Co, LTD, Beijing, China) and GDF15 (Quantikine ELISA Human GDF15, DGD150; R&D Systems, Minneapolis, MN, USA), in plasma from patients were measured using commercially available ELISA kits in accordance with the manufacturer’s instructions.

### 2.3. Total mRNA and Protein Isolation from Tissue

To isolate total RNA from human aortic samples, the RNeasy Fibrous Mini Kit (Qiagen, Venlo, The Netherlands) was used following the manufacturer’s instructions and RNA integrity was determined and quantified by a NanoDrop Spectrophotometer (Thermo Scientific). Protein lysates from human aortic samples were homogenized using a Tissue RuptorII (Qiagen) in a RIPA buffer (150 mM NaCl, 1% (*v/v*) Triton X-100, 0.5% 113 (*w/v*) sodium deoxycholate, 0.1% (*w/v*) SDS, 2 mM EDTA and 50 mM Tris-HCl pH 8) following a standard protocol.

### 2.4. Quantitative Real-Time PCR

DNase I-treated total RNA (1 μg) was reverse transcribed into cDNA using the High Capacity cDNA Archive Kit (Applied Biosystems, Foster City, CA, USA). The mRNA levels were quantified by using specific primers and probes for human CD36 (Hs00169627_m1) and CD38 (Hs01120071_m1) provided by the assay-on-demand system (Applied Biosystems). As housekeeping genes, glyceraldehyde 3-phosphate dehydrogenase (GAPDH; Hs02758991_g1) and β-actin (Hs99999903_m1) were used. Each sample was amplified in duplicate by quantitative RT-PCR in an ABI PRISM 7900HT Sequence Detection System (Applied Biosystems) and the same results were obtained after normalization to both housekeeping genes. Relative mRNA levels were determined using the 2^−∆∆Ct^ method.

### 2.5. Immunostaining and Histology

Tissue specimens were formalin fixed and paraffin embedded. Immunostaining assays were performed in human aortic sections as previously described [24]. For immunohistochemistry assays sections (5 μm) of aneurysmal aortas and donor abdominal aortas were deparaffinized in xylene, rehydrated in graded ethanol and treated with 0.3% hydrogen peroxide for 30 min to block peroxidase activity. Then, sections were blocked with 10% of fetal bovine serum in PBS containing 0.1% Triton X-100and incubated with antibodies against CD3 (SK202, Dako), CD68 (M0876, Dako), CD19 (F0768, Dako), Neutrophil Elastase (M0752, Dako), IgG (A0423, Dako), CD38 (NBP1-47462), GDF15 (NBP1-81050), S100A4 (NBP1-89402) and CD36 (NB400-144) overnight at 4 °C. After incubation with primary antibodies, three washes with PBS were performed to remove excess of unbound antibody and the slides were incubated with a biotinylated secondary antibody (Vector Laboratories, Peterborough, UK) for 1 h. After rinsing 3 times in PBS, standard Vectastain (ABC) avidin-biotin peroxidase complex (Vector Laboratories) was applied to sections for 30 min and after adding the substrate 3,3′-diaminobenzidine (DAB), color was developed. Finally, sections were counterstained with hematoxylin, dehydrated and cleared before mounting.

For antigen colocalization studies, after deparaffinization, antigen retrieval and permeabilization with PBS-0.3% Triton X-100, sections were blocked with PBS containing 5% albumin and 5% FBS for 1 h at room temperature. Double-fluorescence immunostaining was performed by sequentially incubating the sections with mouse polyclonal antibodies against CD206 (ab64693) or CD80 (ab254579) at 4 °C overnight followed by the incubation with a rabbit polyclonal antibody against CD68 (M0876, Dako). After washing the excess of unbound primary antibodies, secondary antibodies (donkey anti-rabbit IgG conjugated to Alexa Fluor 555 and goat anti-mouse IgG conjugated to Alexa Fluor 488, Molecular Probes, Life Technologies) were applied for 1 h at 20–22 °C. Anti-DNA/RNA Damage mouse monoclonal antibody (ab62623) to detect Oxo-8-dG (8-Oxo-7,8-dihydro-2′-deoxyguanosine) was also used followed by an incubation with a secondary antibody goat antimouse conjugated to Alex FLuor 488. Finally, the slides were mounted with ProLong Gold antifade reagent with DAPI (Molecular Probes, Life Technologies Co., Eugene, OR, USA). For negative controls the primary antibody was omitted. Imaging of epifluorescent stainings was performed using a Leica DM6000B microscope, and images were analyzed using Adobe Photoshop CS5 and ImageJ (NIH). Results were quantified as a positive cell number per area in independent sections of AAA.

### 2.6. Reactive Oxygen Species (ROS) Quantification in Plasma

2-hydroxyethidium (2-EOH) was determined in plasma by HPLC with fluorescence detection as described by Laurindo et al. 2008 [25]. Briefly, 25 μL of plasma in the presence of dihydroethidine (DHE, Sigma-Aldrich, St. Louis, MO, USA) 0.8 μM were incubated for 1 h in PBS and centrifuged at 10,000× *g* for 5 min. The supernatant was injected into the chromatographic system (mobile phase: 65% water with 0.3% Trifluoracetic acid: 35% acetonitrile; flow 1 mL /min; column kromasil C18, 5 μm, 200 × 4.6 mm-Teknokroma Analitica; excitation 510 nm and emission 595 nm). Xanthine/xanthine oxidase (XO) was used to calibrate the signal of O^2−^ and the calibration curve was constructed by comparing the production of 2-EOH and the ratio of XO activity/HE concentration (85–605 nU XO/ng HE). 2-EOH present in the samples was quantified by comparing with the calibration curve based on the reaction xanthine–xanthine oxidase in the presence of DHE (Sigma-Aldrich, St. Louis, MO, USA) following the method described by Michalski et al. 2014 [26]. 

### 2.7. Western Blot

Aortic tissue lysates were separated by SDS-PAGE, transferred to the PVDF blotting membrane (Immobilon, Millipore. Merck KGaA, Darmstadt, Germany) and incubated overnight at 4 °C with an antibody against S100A4 acquired from Novus Biologicals (Bio-Techne LD-R&D Systems Europe Ltd., Abingdon, UK). Equal loading of protein in each lane was verified by β-actin (A5441, Sigma Aldrich, St. Louis, MO, USA).

### 2.8. Finite Element Analysis

Finite element analysis was performed by using A4clinics-Research Edition software (VASCOPS Vascular Diagnosis Company, Graz, Austria) on the computed tomography angiography (CTA) of 60 of the AAA patients included in the present study. Only one member of our group (B.S.) performed the analysis to avoid inter-observer errors as previously described [27]. The three dimensional AAA geometry was acquired from routine CTA imaging data. The resultant geometry is subdivided into multiple contiguous elements that form a fine mesh. The lumen, intraluminal thrombus and external wall data were acquired separately and semi-automatically. Manual correction is allowed by the program if some special point is found, such as a penetrating ulcer or some other unusual anatomy. Wall stress computation of the AAA can be obtained after providing the appropriate material properties and components of the aneurysmal wall and results in aneurysm specific wall stress distribution. We additionally analyzed the segment from the infrarenal aorta to the iliac bifurcation. Among the biomechanical variables that were determined, PWS (the maximal stress on the surface of the AAA wall based on aneurysm shape, diameter and blood pressure values) was the variable chosen for this study.

### 2.9. Statistical Analysis 

Results are expressed as mean ± SEM of the number (*n*) of samples indicated in the figure legends. Demographic and clinic characteristics of the human individuals were expressed as mean ± SD. Normal distribution of the variables was determined by Shapiro–Wilk test. When data followed a normal distribution, differences between two groups were assessed using the Student’s *t* test (two-tailed). When normality failed, the Mann–Whitney rank sum test was applied to compare two groups. To study the association between variables following a normal distribution, Pearson product-moment correlation coefficient was used whereas Spearman’s rank-order correlation was applied to determine the association between variables that are not.

To assess potential confounding factors (age, sex, hypertension, DM and smoking), multivariate logistic regression analysis was performed after log-transforming non-normal distributed variables using the statistical software package R-3.2.2 (www.r-project.org (accessed on 22 March 2021)). The results were expressed as an odd ratio (OR) and 95% confidence intervals (CIs). Data analysis was carried out using GraphPad Prism version 8.4.2 software (La Jolla, CA, USA). Values of *p* ≤ 0.05 were considered significant.

## 3. Results

### 3.1. Immune Infiltrate and Immunoglobulins in Human AAA

**Figure 1:**
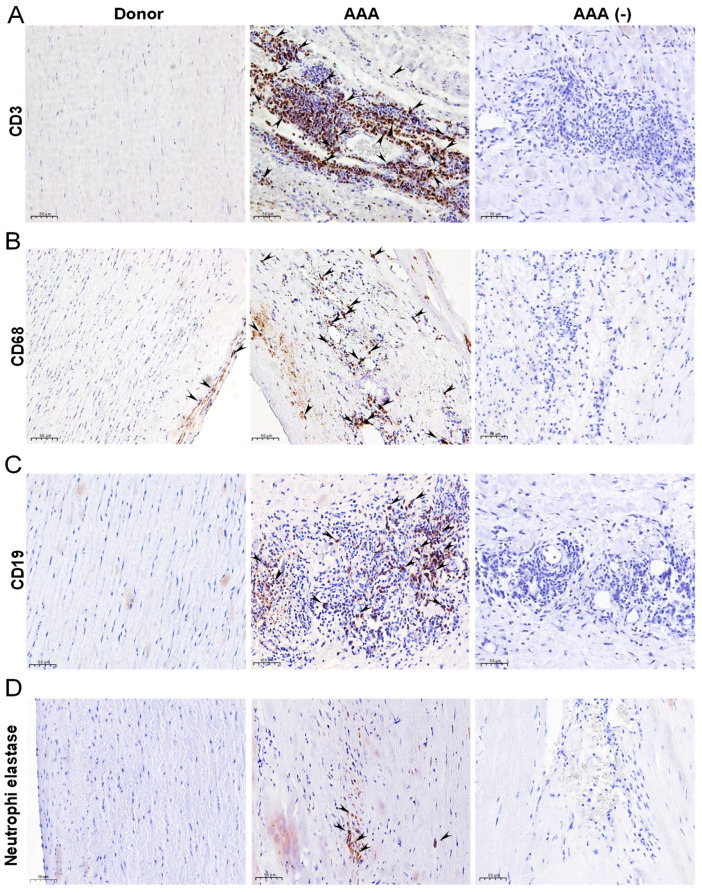
Immune infiltrate in AAA. (**A**–**D**) Representative images of immunostaining assays performed in abdominal aorta sections from AAA patients and donors targeting CD3 (T-lymphocyte s), CD68 (macrophages), CD19 (B-lymphocytes) and elastase (Neutrophils), respectively (*n* = 10; Scale bars: 50 µm). AAA (-) indicates negative control for immunohistochemistry. Arrows indicate the positively stained cells.

We evaluated the immune cell content in aneurysmal tissue from our AAA patient cohort. Table 1 shows demographic data of the patients and donors included in this study. The majority of the immune cells present in aneurysmal wall were T-cells (CD3), macrophages (CD68) and B cells (CD19), and to a lesser extent neutrophils (neutrophil elastase) as shown in Figure 1A–D.

**Figure 2:**
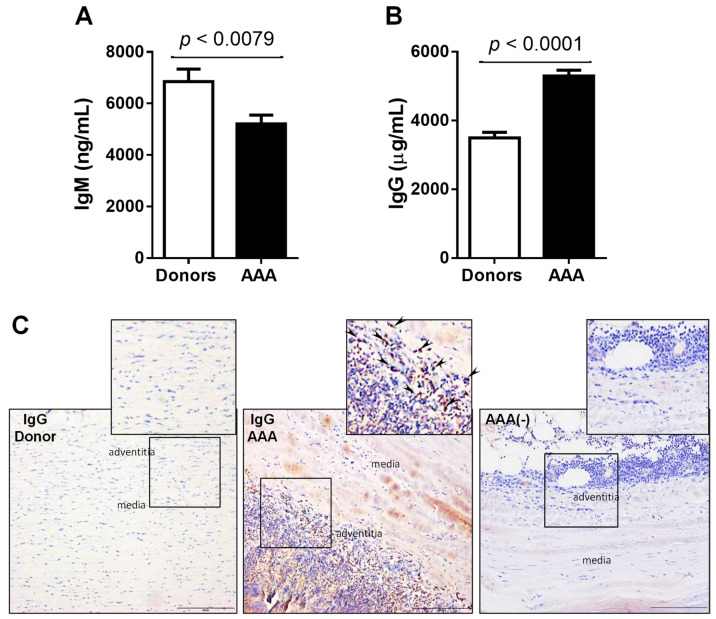
Circulating levels of IgM and IgG are altered in AAA patients. (**A**,**B**) Circulating levels of IgM and IgG, respectively in AAA (*n* = 94) vs. healthy donors (*n* = 46). (**C**) Representative images of immunostaining assays performed in abdominal aorta sections from donors and AAA patients targeting IgGs (*n* = 10; scale bars: 100 µm). AAA (-) indicates negative control for immunohistochemistry. Arrows indicate the positively stained cells.

Recent studies have shown that there are distinct macrophage subsets with different functionalities in human and experimental AAA tissues [9,28,29]. Aneurysmal tissue from AAA patients exhibited markers of both classical M1 (CD68+ CD80+) macrophages and alternatively activated M2 (CD68+ CD206+) macrophages (Supplementary Figure S1A,B) in the immune infiltrate. On another hand, we found that circulating IgM levels were significantly decreased, whereas IgG levels were increased in AAA patients compared to controls (Figure 2A,B). Confounding factors had no impact on the difference between circulating levels of IgGs or IgMs found in patients with AAA in comparison with those found in control subjects (Table S1). When IgG was measured in situ in paraffin-embedded tissue, a higher number of positively stained cells located in the adventitia was observed in stark contrast to that observed in healthy aortas (Figure 2C).

### 3.2. Vascular and Circulating Levels of Oxidative Stress and Inflammation-Related Biomarkers in AAA Patients

**Figure 3:**
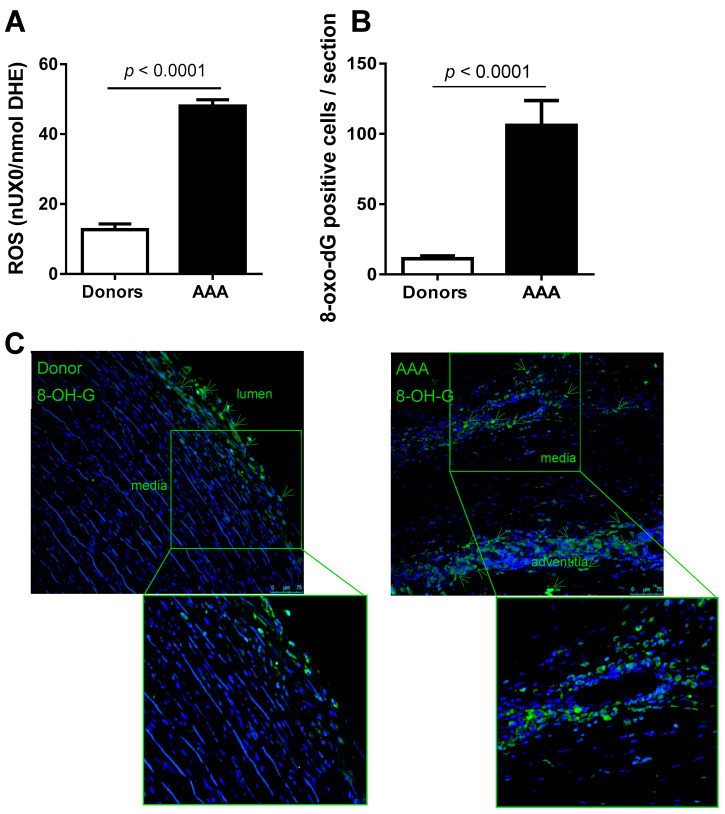
Reactive oxygen species (ROS) circulating levels are increased in AAA patients. (**A**) Quantification of ROS plasma levels in AAA (*n* = 94) vs. healthy donors (*n* = 46). (**B**) Histograms showing the quantification of the number of positive cells per aortic area (10×). Results are expressed as mean ± SEM. (**C**) Representative images of immunostaining assays performed in abdominal aorta sections from donors and AAA patients targeting 8-oxo-DG positive stained cells.

When ROS plasma levels were tested in our cohort, we observed a 2.5-fold increase in AAA patients compared to controls (Figure 3A) and this difference remained significant after adjusting for confounding factors (Table S1). This was accompanied by an increase in the number of positive cells for 8-Oxo-7,8-dihydro-2′-deoxyguanosine (a marker of DNA damage due to ROS) in aneurysmal tissues compared to donor aortas (Figure 3B,C).

**Figure 4:**
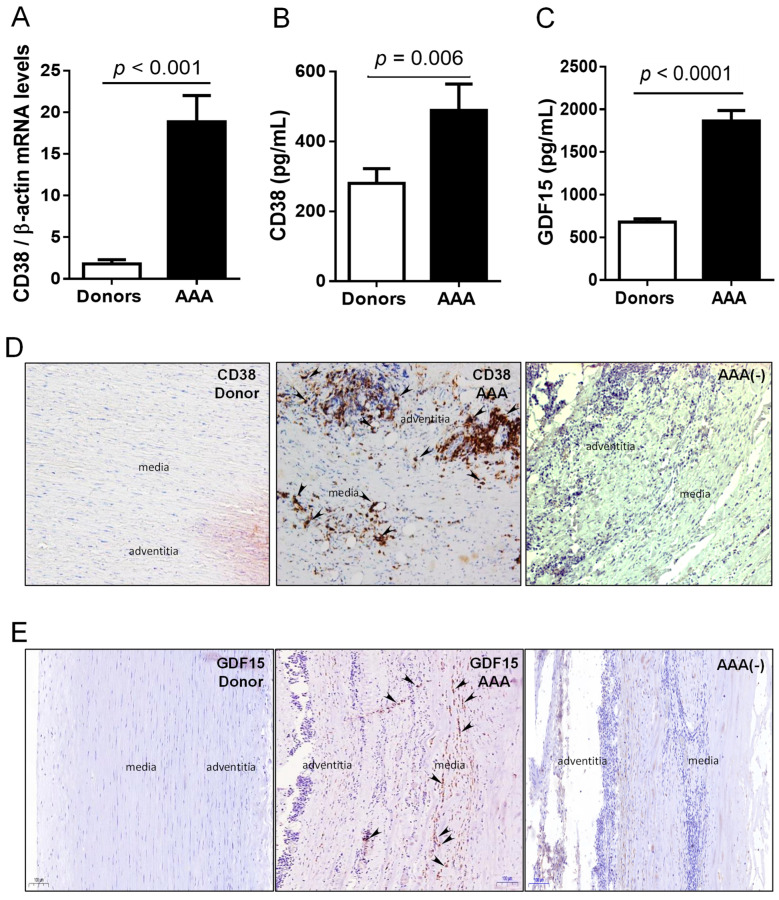
CD38 and GDF15 expression and circulating levels are increased in AAA patients. (**A**) Human abdominal aortic mRNA levels of CD38 measured by quantitative real-time PCR and normalized to β-actin in healthy donors (*n* = 15) and patients (AAA) (*n* = 80). (**B**) CD38 plasma levels in AAA (*n* = 94) vs. healthy donors (*n* = 46); (**C**) plasma levels of GDF15 in AAA (*n* = 94) vs. healthy donors (*n* = 46). (**D**) Representative images of immunostaining assays performed in abdominal aorta sections from donors and AAA patients targeting CD38 (*n* = 10; scale bars: 100 µm). (**E**) Representative images of immunostaining assays performed in abdominal aorta sections targeting GDF15 (*n* = 0; scale bars: 100 µm). AAA (-) indicates negative control for immunohistochemistry. Arrows indicate the positively stained cells.

CD38 is expressed on the surface of lymphocytes, macrophages and neutrophils, and it is involved in their activation, proliferation and homing by interaction with CD31 in endothelial cells and in the regulation of the innate immune response [14,17,18]. It has been recently involved in the production of O^2−^ and in the modulation of Nrf2 activity in VSMC [30]. Hematopoietic and vascular cells also express CD36 receptor in response to oxidized lipids [23] and CD36 signaling downregulates key antioxidant factors, leading to the accumulation of ROS [31]. CD38 and CD36 mRNA levels were upregulated in the aneurysmal vascular wall compared to healthy abdominal aortas (Figure 4A, Supplementary Figure S2A). Immunohistochemistry revealed that CD38 and CD36 were barely detectable in donor aortas, but in aneurysmal tissues CD38 was markedly found in the media and the adventitia layers (Figure 4D) whereas CD36 was mainly observed in the media (Supplementary Figure S2B). Regarding plasma levels, we found a 1.7-fold increase of CD38 in AAA patients compared to controls (Figure 4B) that remained significant after adjusting for confounding factors (Table S1). However, the circulating levels of soluble CD36, which were barely detected or undetected in most of the analyzed samples, were similar in both groups (Supplementary Figure S2C).

GDF15 is a stress-induced cytokine, produced and secreted by endothelial cells, macrophages, VSMC and cardiac myocytes in response to ischemia, proinflammatory stimuli or oxidative stress [19,20]. Plasma GDF15 levels were increased by 3-fold in AAA patients compared to controls (Figure 4C) and this difference remained significant after adjusting for confounding factors (Table S1). GDF15 was slightly expressed in the media layer of aneurysmal aortas, whereas no expression was detected in healthy vessels (Figure 4E).

**Figure 5:**
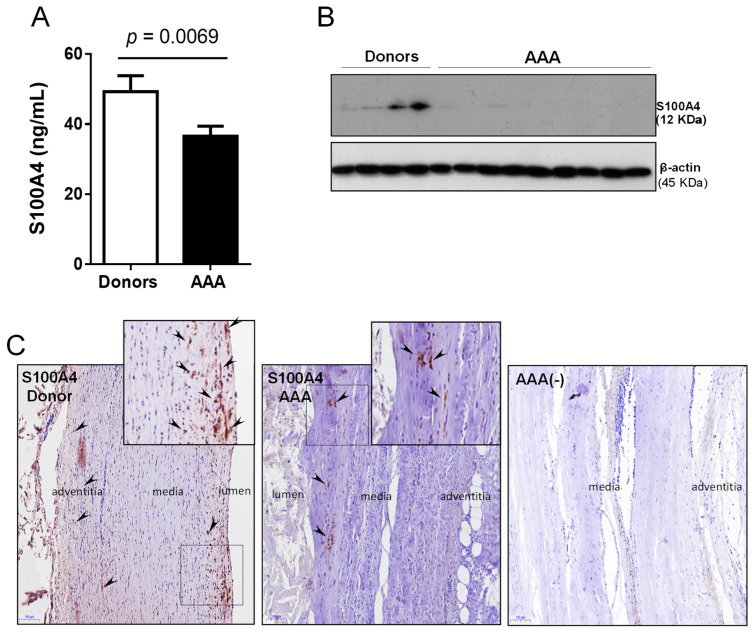
S100A4 is decreased in AAA patients compared to healthy donors. (**A**) Plasma levels of S100A4 in AAA (*n* = 94) vs. healthy donors (*n* = 46); (**B**) representative Western blot analysis of S100A4 in protein lysates of abdominal aortas from AAA patients and donors (AAA: *n* = 15 and donors: *n* = 10); (**C**) representative images of immunostaining assays performed in abdominal aorta sections from donors and AAA patients targeting S100A4 (*n* = 10; scale bars: 100 µm). AAA (-) indicates negative control for immunohistochemistry. Arrows indicate the positively stained cells.

S100A4 is a potent trigger of inflammatory processes by inducing the release of cytokines, growth factors and ROS production under different pathological conditions [22,32]. Circulating S100A4 was significantly decreased in AAA patients compared to controls (Figure 5A) and this difference remained significant after adjusting for every confounding factor (Table S1). This was supported by a decreased S100A4 expression in the aneurysmal wall compared to healthy abdominal aortas (Figure 5B,C). 

### 3.3. Correlation of Biomarker Levels and Preoperative Aortic Diameter

**Figure 6:**
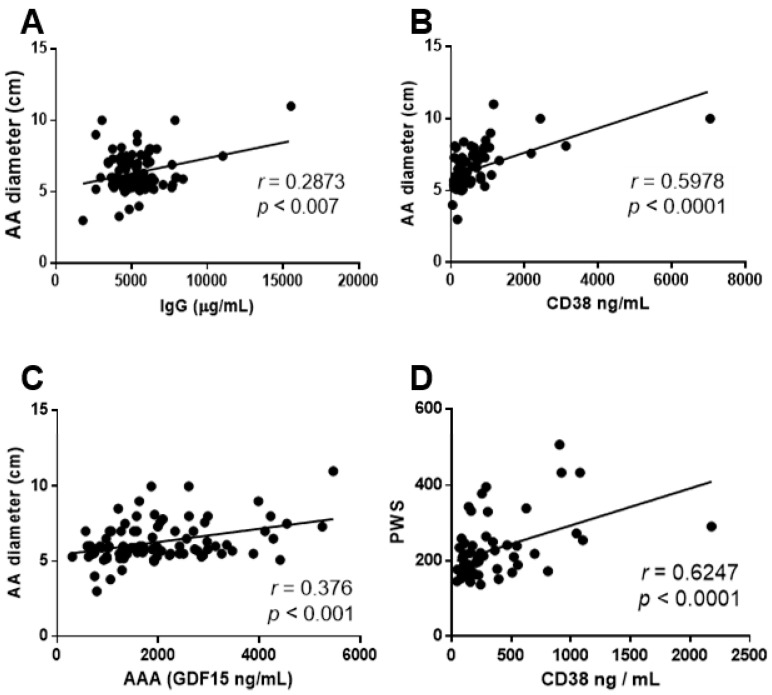
IgG, CD38 and GDF15 circulating levels positively correlate with the AAA diameter whereas only CD38 correlates with PWS. (**A**) Graphs showing the correlation analysis between IgG plasma levels and AAA diameter (*n* = 90) and (**B**,**C**) the correlation analysis between CD38 plasma levels and AAA diameter (*n* = 90) or PWS values in AAA patients (*n* = 58). (**D**) Graph showing the correlation analysis between GDF15 plasma levels and AAA diameter (*n* = 90). The r and *p*-values are obtained by performing the Spearman or the Pearson correlation coefficient test. Results are expressed as mean ± SEM.

Positive correlations between IgG, GDF15 or CD38 levels and preoperative abdominal aortic diameter were found by correlation analysis (Figure 6A–C) whereas no correlations between the diameter and circulating ROS or S100A4 levels were found (data not shown). When correlations between each biomarker and PWS were analyzed, only a positive correlation with CD38 levels was found (Figure 6D). All the established associations were maintained after adjustment for age.

## 4. Discussion

The identification of novel circulating markers of AAA is an active focus of investigation. In the present study, we aimed to determine the value of circulating levels of immune-inflammatory and oxidative stress-related markers to assist AAA management. Our results showed higher IgG, ROS, CD38, CD36 and GDF15 expression in aneurysmal tissue of AAA patients, and higher circulating levels of soluble IgG, ROS, CD38 and GDF15 compared to controls. In contrast, IgM and S100A4 were found decreased in AAA patients while no differences were found in soluble CD36 levels between AAA and controls. When we studied the association between circulating levels of all the altered molecules and the preoperative diameter of the abdominal aorta of AAA patients, only IgG, CD38 and GDF15 showed a significant correlation independently of age and of cardiovascular risk factors such as hypertension or diabetes. PWS, evaluated using computer modeling by finite element analysis, is a useful parameter for predicting the risk of rupture [33,34]. Interestingly, when we studied the association of the different circulating molecules with the PWS values, only CD38 was positively correlated.

According to our data, circulating IgM, ROS and S100A4 did not correlate either with the diameter or with PWS and since CD36 circulating levels are barely detected, no correlations were studied. However, we cannot underestimate their role in the pathogenesis of AAA. In fact, human and animal studies have confirmed that oxidative stress is well associated with AAA development [13,35,36,37]; whereas other authors have previously reported CD36 overexpression in aneurysmal aortas [38], and found a positive correlation between the amount of CD36-labeled platelets in the blood of AAA patients and aneurysm diameter [38,39]. Regarding S100A4, circulating levels and tissue expression were strikingly decreased in both plasma and aortic samples from AAA patients compared to controls. Since immune-histochemical studies in donor aortas localize S100A4 expression mainly in VSMC, its decrease in AAA could be due to the well-reported loss of VSMC in this disease. On another hand, despite a previous study reported an increase of S100A4 levels in tissue and in serum of patients with thoracic aortic aneurysm [40], it could be due to the different mechanisms underlying the pathogenesis of abdominal and thoracic aneurysms development.

In our AAA patient cohort the circulating levels of IgM were decreased, of note in other cardiovascular diseases IgM have been described as protective [41,42,43], so further studies are needed in order to clarify if this will be the case in AAA. On the contrary, we found an increase in IgG levels both systemically and in the aneurysmal tissue, potentially indicating an activation of long-lived plasma B cells [44] and of B cells residing in the aneurysmal tissue respectively [8,10]. Immunoglobulins are deposited on AAA tissue and mostly localized to adventitial cells suggesting that they may impact on diverse cell types in the aneurysmal wall [45]. Accordingly, we found increased deposits of IgG and a high number of B-cells in aneurysmal tissue together with T-lymphocytes and macrophages, which are most of the cells detected in the inflammatory infiltrate of the aneurysmal wall. The main characteristic of M1 macrophages is the production of proteolytic enzymes and proinflammatory cytokines. In contrast to M1, M2 macrophages participate in the anti-inflammatory response and are involved in ECM remodeling and tissue repair [28,29]. Interestingly, the number of M2 macrophages was higher in AAA to that number of M1-like macrophages indicating that the first ones are quite abundant in advanced AAA disease. This is in agreement with previous studies where a higher M2 macrophages cellularity in aneurysmal tissue has been reported [46]. Further, Sharma et al. recently demonstrated that facilitating the recruitment of M2-like macrophages promoted formation of AAA counteracting the concept that M2 macrophages are beneficial in AAA [47]. Interestingly, blocking oxidative metabolism drives the macrophages toward an M1-like state and impairs the development of an M2-like phenotype. Thus, we hypothesized that the high concentration of ROS in diseased tissue and blood found in our AAA patients cohort might influence the M1/M2 polarization ratio as previously described [48,49].

We additionally found that the circulating levels of other biomarkers such as the transmembrane enzyme CD38 and GDF15 were increased in plasma of AAA patients. Moreover, CD38 was highly expressed in aortic aneurysm tissue. In agreement, a recent study has proposed the implication of CD38 signaling as a proinflammatory intermediate in AAA [50]. Due to the CD38 relationship with the modulation of immune response, aging and oxidative damage in vascular cells [51], we believe that the exacerbated expression of CD38 found in the aneurysmal arterial wall might contribute to maintain chronic inflammation, ROS generation and VSMC apoptosis during AAA development. Further studies are needed to clarify the mechanisms by which CD38 is involved in this disease. Furthermore, accumulating evidence suggests that circulating GDF-15 concentrations are elevated and serve as independent prognostic biomarker in a wide spectrum of cardiovascular diseases, including ischemic heart disease, heart failure, atherosclerosis, hypertrophic cardiomyopathy and as a predictor of first-ever stroke in hypertensive patients [52,53,54,55]. 

Finally, we would like to remark the limitations of the work with human samples. The availability of aortic specimens from multi-organ donors was limited by the age and sex requirements. Moreover, it was not possible to obtain blood samples from these donors and for this reason, healthy abdominal aorta and blood samples taken as controls came from two different groups of individuals. Regarding AAA patients data, we included in Table 1 those parameters available in their clinical reports, and the average of PWS values calculated with the finite element analysis from the available CTAs (*n* = 60) of the studied cohort.

## 5. Conclusions

In summary, our study shows the increase of vascular and circulating levels of IgG, ROS, CD38 and GDF15 in patients with AAA that could contribute to the chronic inflammatory state of this disease. Most importantly, our findings suggest the potential usefulness of the circulating levels of IgG, CD38 and GDF15 to assist AAA management in conjunction with aneurysm diameter and/or PWS during the follow-up of these patients.

## Figures and Tables

**Table 1:** Demographics of individuals included in the study.

	mRNA		Plasma	
	AAA	Normal Aorta	AAA	Blood Donors
**N**	80	15	94	46
**Age (years)**	70 ± 6.2	63 ± 11.5	71.4 ± 6.5	59.9 ± 5.3 *
**Women (%, *N*)**	5 (4)	20 (3)	4.3 (4)	10.9 (5)
**Aortic diameter (mm)**	63.5 ± 1.4	-	63.9 ± 1.4	-
**Dyslipidemia (%, *N*)**	56.3 (45)	26.7 (4)	63.8 (60)	-
**HTN (%, *N*)**	81.25 (66)	40 (6)	77.6 (73)	19.6 (9)
**Diabetes (%, *N*)**	21.25 (17)	33.3 (5)	19.1 (18)	23.91 (11)
**Smokers/ex-smokers (%, *N*)**	81.25 (66)	40 (6)	80.1 (76)	45.7 (21)
**PWS (kPa)** **TC (mM)**	222 ± 74.8 -	- -	222 ± 74.8 3.9 ± 1.2	- 5.6 ± 0.8
**HDL-C (mM)**	-	-	1 ± 0.3	1.5 ± 0.3
**LDL-C (mM)**	-	-	2.3 ± 0.9	3.5 ± 0.7
**VLDL-C (mM)**	-	-	0.6 ± 0.3	0.6 ± 0.3
**Triglycerides (mM)**	-	-	1.3 ± 0.7	1.4 ± 0.7

Nominal variables are presented as %. Continuous variables are presented as mean ± SD. Due to the nature of normal aorta and plasma samples from donors, some of the clinical characteristics are not always recorded and infra-evaluation of them is probable. HTN, chronic hypertension; PWS, peak wall stress; TC: total cholesterol; HDL-C: high-density lipoprotein-cholesterol; LDL-C: low density lipoprotein-cholesterol. * *p* < 0.05 AAA vs. Donors.

## Data Availability

The data used to support the findings of this study is available from the corresponding author upon request.

## Supplementary Materials

The following are available online at https://www.mdpi.com/article/10.3390/antiox10040602/s1, Figure S1: Presence of M1 and M2 infiltrated macrophages in AAA tissue, Figure S2: CD36 protein is increased in aneurysmal tissue but not in the circulation; Table S1: Logistic regression analysis.
