# Supplementary material for: Oxidative Stress and Inflammatory Markers in Abdominal Aortic Aneurysm
Source: Antioxidants (Basel). 2021 Apr 14;10(4):602. doi: 10.3390/antiox10040602 (PMC8070751; doi:10.3390/antiox10040602)
Supplement: Supplementary file 1 [file antioxidants-10-00602-s001.pdf]

SUPPLEMENTARY MATERIAL

Oxidative stress and inflammatory markers in abdominal aortic aneurysm.

Running title: Novel markers for AAA

Authors: David Sánchez-Infantes[1,2], Meritxell Nus[3,4], Miquel Navas-Madroñal[5,6], Joan Fité[7], Belén Pérez[8], Antonio J. Barros-Membrilla[9], Begoña Soto[7], José Martínez-González[4,10], Mercedes Camacho[4,5,6], Cristina Rodriguez[4,5,6], Ziad Mallat[3], María Galán [4,5,6].

[1]Department of Basic Sciences of Health, Area of Biochemistry and Molecular Biology, University Rey Juan Carlos , Alcorcón, Spain.

[2]Centro de Investigación Biomédica en Red-Fisiopatología de la Obesidad y Nutrición (CIBEROBN), ISCIII, Madrid, Spain.

[3]Division of Cardiovascular Medicine, University of Cambridge. Cambridge, United Kingdom.

[4]Centro de Investigación en Red de Enfermedades Cardiovasculares (CIBERCV), Madrid, Spain.

[5]Institut de Recerca del Hospital de la Santa Creu i Sant Pau, Barcelona, Spain. 08041.

[6]Instituto de Investigación Biomédica Sant Pau (IB Sant Pau), Barcelona, Spain.

[7]Servicio de Angiología, Cirugía Vascular y Endovascular, Hospital de la Santa Creu i Sant Pau. Barcelona, Spain.

[8]Faculty of Medicine, Universidad Autónoma de Barcelona, Bellaterra. Barcelona, Spain.

[9]Unidad Funcional de Patología de la Aorta (UPA), Servicio de Cardiología, Hospital de la Santa Creu i Sant Pau. Barcelona, Spain.

[10]Instituto de Investigaciones Biomédicas de Barcelona (IIBB-CSIC), IIB Sant Pau, Barcelona, Spain.

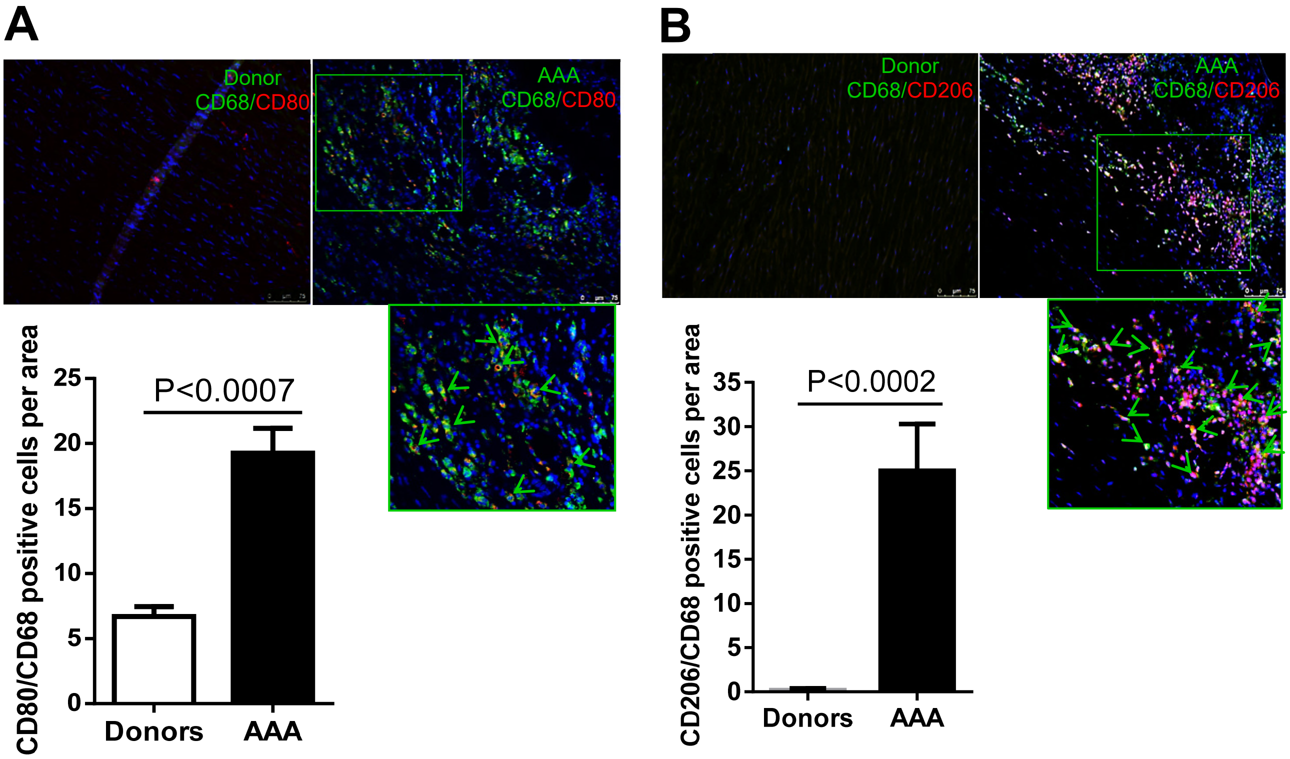

Figure Supplementary 1. Presence of M1 and M2 infiltrated macrophages in AAA tissue. A-B) Representative images of immunostaining assays performed in abdominal aorta sections from donors and AAA patients targeting M1 (CD68 CD80) and M2 (CD68 CD206) macrophages (n=10). The histograms below show

the quantification of the number of positive cells per aortic area (10x). Results are expressed as mean ± SEM.

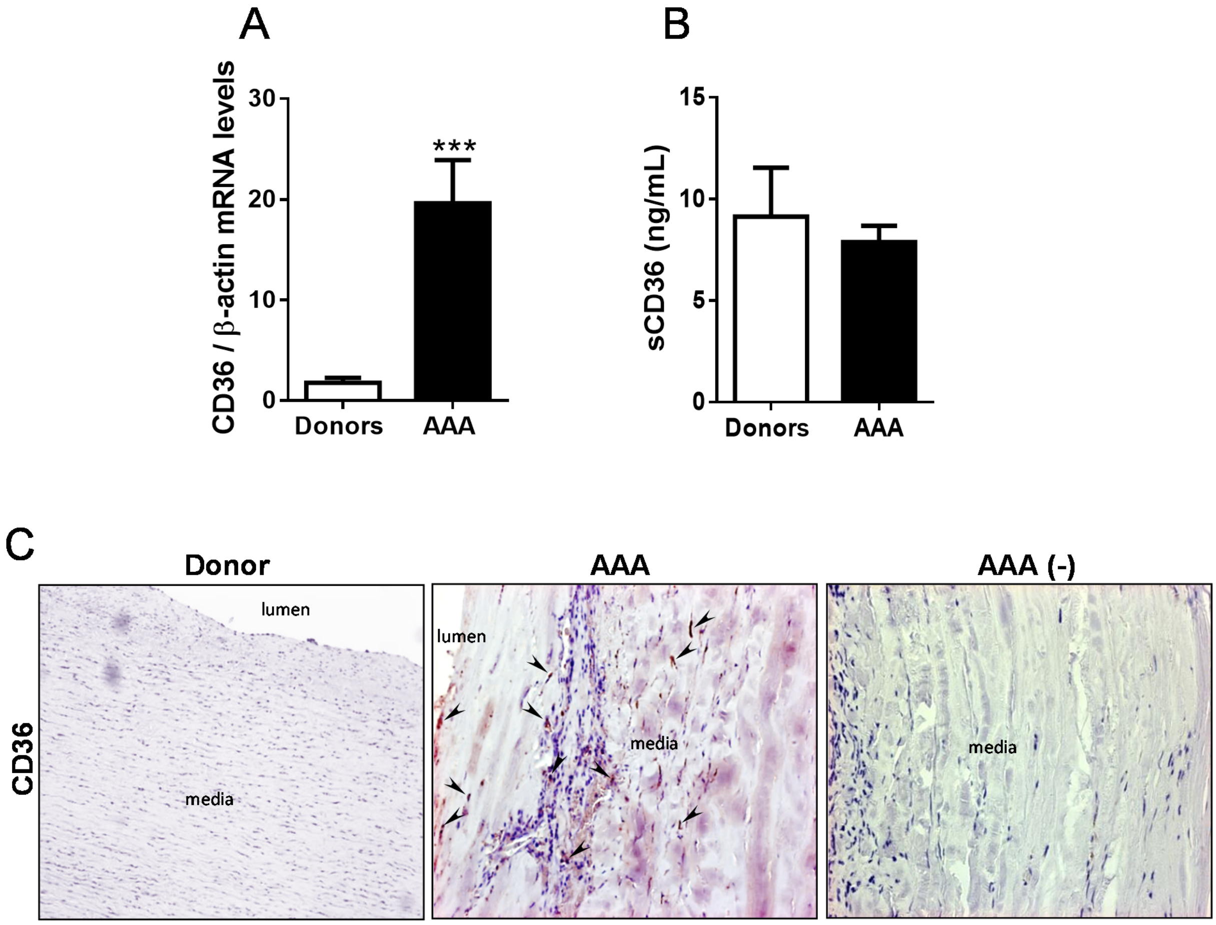

Figure Supplementary 2. CD36 protein is increased in aneurysmal tissue but not in the circulation. A) Human abdominal aortic mRNA levels of CD36 measured by quantitative real Time PCR analysis and normalized to β-actin in healthy donors (n=15) and patients (AAA) (n=80); **B)** Plasma levels of CD36 in AAA (n=32) vs healthy donors (n=21); **C)** Representative images of immunostaining assays performed in abdominal aorta sections from donors and AAA patients targeting CD36 (n=10; Scale bars: 100 μm). Arrows indicate the positively stained cells. AAA (-) indicates negative control for immunohistochemistry. Results are expressed as mean ± SEM. *** $p<0.001$ *vs.* AAA.

Supplementary Table S1. Logistic regression analysis.

	Covariate	OR	95 % C.I. for EXP (B) Lower	Upper	P value
Unadjusted	IgG	28.007	7.529	104.187	< 0.0001*
Adjusted for age		20.863	3.719	117.028	0.00045*
Adjusted for sex		27.185	7.211	102.483	< 0.0001*
Adjusted for HTA		12.861	3.206	51.589	< 0.0001*
Adjusted for DM		28.696	7.581	108.625	< 0.0001*
Adjusted for smoking		25.944	6.443	104.467	< 0.0001*
Adjusted for all factors		8.221	0.670	100.951	0.043*
Unadjusted	IgM	0.096	0.020	0.454	0.003*
Adjusted for age		0.018	0.001	0.254	0.004*
Adjusted for sex		0.097	0.020	0.478	0.004*
Adjusted for HTA		0.143	0.021	0.987	0.048*
Adjusted for DM		0.1	0.021	0.987	0.004*
Adjusted for smoking		0.068	0.012	0.408	0.003*
Adjusted for all factors		0.008	0.0001	0.578	0.027*

Unadjusted	ROS	1.314	1.168	1.466	< 0.0001*
Adjusted for age		1.816	0.999	3.301	0.050*
Adjusted for sex		1.376	1.197	1.582	< 0.0001*
Adjusted for HTA		1.495	1.137	1.966	0.004*
Adjusted for DM		1.324	1.179	1.488	< 0.0001*
Adjusted for smoking		1.348	1.180	1.540	< 0.0001*
Adjusted for all factors		1.708	1.012	2.884	0.989
Unadjusted	CD38	6.447	2.201	18.885	0.001*
Adjusted for age		6.927	2.058	23.315	0.002*
Adjusted for sex		6.617	2.247	19.485	0.001*
Adjusted for HTA		6.361	1.850	21.879	0.003*
Adjusted for DM		6.619	2.205	19.869	0.001*
Adjusted for smoking		7.188	2.274	22.728	0.001*
Adjusted for all factors		10.119	1.813	56.485	0.008*
Unadjusted	GDF15	24.653	9.215	65.957	< 0.0001*
Adjusted for age		13.755	4.587	41.248	< 0.0001*
Adjusted for sex		20.749	7.696	55.942	< 0.0001*
Adjusted for HTA		18.903	6.239	57.275	< 0.0001*
Adjusted for DM		24.721	9.191	66.490	< 0.0001*
Adjusted for smoking		24.608	8.653	69.982	< 0.0001*
Adjusted for all factors		5.793	1.767	18.993	0.004*
Unadjusted	S100A4	0.093	0.025	0.344	0.0004*
Adjusted for age		0.094	0.020	0.350	0.020*
Adjusted for sex		0.091	0.024	0.350	0.0005*
Adjusted for HTA		0.114	0.025	0.521	0.005*
Adjusted for DM		0.094	0.025	0.350	0.0004*
Adjusted for smoking		0.079	0.018	0.341	0.001*
Adjusted for all factors		0.112	0.007	1.747	0.118

OR: Odd Ratio; 95% CI: 95 confidence interval. *P≤0.05 vs. Donors: statistically significant.
